# The relationship between resilience and mental health: mobile phone dependence and its differences across levels of parent-child conflict among left-behind adolescents: a cross-sectional network analysis

**DOI:** 10.1186/s12889-025-22105-8

**Published:** 2025-03-10

**Authors:** Xiaoya Yuan, Yaxin Mao, Xiaomin Xu, Ruolan Peng, Min Tang, Gang Dai, Xinyi Tang, Haojie Fu, Xiao Zhong, Guanzhi Zhang, Bin Wang

**Affiliations:** 1 Law School of Southwest University of Science and Technology, No.59, QingLong Avenue Fucheng District, Mianyang, Sichuan 621010 China; 2 Psychosocial Service and Crisis Intervention Research Center, Southwest University of Science and Technology, East Building7-409, No. 59 of Qinglong Street, Fucheng District, Mianyang, Sichuan Province 621010 China; 3 School of Foreign Languages and Cultures, Southwest University of Science and Technology, East Building7-409, No. 59 of Qinglong Street, Fucheng District, Mianyang, Sichuan Province 621010 China; 4 Shanghai Research Institute for Intelligent Autonomous Systems, Tongji University, Shanghai, China; 5 School of Psychology, Beijing University of Sports, Beijing, China

**Keywords:** Left-behind adolescents, Resilience, Mental health, Mobile phone dependence, Parent–child conflict, Network analysis

## Abstract

**Background:**

Mobile phone dependence and mental health problems have become increasingly prominent among left-behind adolescents in China. In recent years, some studies have focused on the important role of parent–child relationship and psychological resilience. Therefore, this study aims to explore the multidimensional relationships among resilience, mental health, and mobile phone dependence among left-behind adolescents, and to assess the impact of parent–child conflict level on these relationships.

**Methods:**

The Brief Symptom Inventory (BSI-18), the Chinese version of the Mobile Phone Addiction Index (MPAI), the Resilience Scale for Children and Adolescents (RSCA), and the Parent–Child Conflict Scale were used to investigate 2,100 left-behind adolescents in Sichuan Province, and R was run to make network analysis and network comparison.

**Results:**

(1) A structurally stable network relationship exists between left-behind adolescents' resilience, mental health, and mobile phone dependence; (2) BSI3 (Anxiety) is the most important node of the network model, followed by MPAI1 (the inability to control cravings subscale); (3) MPAI1 (the inability to control cravings subscale) and RSCA4 (family support) are key to connect resilience, mental health, and smartphone addiction in the study sample; (4) There was a significant difference in the network structure between the high- and low-level groups of parent–child conflict, no significant difference in the global strength of the network, and a significant difference in the centrality of strength and the centrality of bridge strength.

**Conclusions:**

Chinese left-behind adolescents' resilience and mental health, mobile phone dependence are both independent and interact with each other to some extent. Specifically, high centrality dimensions such as anxiety, the inability to control cravings, and family support can be prioritised for intervention in related treatments, or reducing parent–child conflict and enhancing resilience to mitigate mobile phone dependence among left-behind adolescents, thus improving their mental health.

**Supplementary Information:**

The online version contains supplementary material available at 10.1186/s12889-025-22105-8.

## Introduction

With China's rapid political and economic development, a large number of people in underdeveloped regions of the central-west have begun to move to the developed regions searching for jobs, which gives rise to the problem of left-behind children due to family, policy, and other factors. As of 2020, China saw 66.93 million left-behind children, and 138 million who were affected by population mobility, accounting for 46.4% of China's total child population [1]. These children, who are left behind are not able to stay with their parents for a long time, lack parental care, are prone to loneliness, anxiety, depression, and other negative emotions, which not only influence their studies and lives, but also may have long-term adverse effects on their mental health. Research has found that, from the past to the present, migrant parents are more likely to hurt their mental health, including emotions and behaviour, and the situation might become worse over time [2]. Meanwhile, these children tend to develop mobile phone dependence [3]. As a means of communication tools provided by parents for their children, smartphones expose children to addiction thanks to being alone for long periods and being spoiled by their elders. This may lead to their indulgence in the virtual world, which may have a negative impact on their social skills, learning, behaviour and mental health [4]. Left-behind adolescents experiencing puberty have rapid physical and mental development and face more severe academic pressure and social environment. Compared with non-left-behind adolescents, left-behind ones show higher risk propensity and prevalence of mental health problems and mobile phone dependence [5]. So we should pay more attention to left-behind youth and provide them with more comprehensive and effective support and guidance.

With the popularity of smartphones, the addictive problem has become a new form of Internet addiction in the mobile era, which is a behavioural addiction that causes psychological and behavioural phenomenon of users due to the misuse of mobile phones [6]. The 44th report of China Internet Network Information Centre shows that the number of mobile phone holders in China has reached 847 million, of which 17% are teenagers aged 10 to 19. Empirical studies in different countries have found that adolescent mobile phone dependence is negatively associated with mental health [7, 8]. Mobile phone dependence and mental health have a very strong relationship, interacting with each other and even risking co-morbidities [9]. The cognitive-behavioural model of pathological Internet use proposed by Davis [10] argues that psychopathologies such as depression, anxiety, and substance dependence are distally necessary causes of pathological Internet use symptoms. According to the compensatory Internet use model proposed by Kardefelt-Winther, negative life situations can increase online behaviour to alleviate negative emotions [11]. Individuals with poor mental health are more vulnerable to negative emotions and behavioural change suffering negative life issues [12], which leads to mobile phone dependence.

Resilience is often described as the ability to revive or overcome certain adversity in order to extract a positive outcome from a negative event or situation [13]. Current research has found that resilience has an important role in the mental health and prevention of mobile phone dependence among left-behind adolescents [14]. A meta-analysis of 25 studies showed that despite differences in research objectives and instruments, higher resilience was associated with fewer mental health problems [15]. At the same time, resilience is also an important predictor of mobile phone dependence, and empirical studies have found that self-resilience related to relationships, curiosity and emotional control have been found to moderate mobile phone use in both men and women [16]. Existing research suggests that resilience can both directly and negatively predict mobile phone dependence among Chinese adolescents [17] and studies from different countries have found that resilience can also serve as a mediator [14] or an adjustment [18] to influence mobile phone dependence tendencies. Meanwhile, mobile phone dependence depletes individuals' self-control ability [19], thus reducing their level of resilience. Richardson's resiliency model views resilience as a dynamic process in which individuals experience disruption, adjustment, integration and reestablishment of equilibrium in stressful situations, protective factors interacts with biological, psychological, and social risk factors to lead to four different adaptive outcomes [20]. Researchers have pointed out that resilience is regarded as one of the key protective elements of Internet addiction, and that Internet addiction often stems from the individual's lack of resilience in self-control and coping with stress and frustration [21]. Mobile phone dependence and Internet addiction are both behavioural addictions with similarities and may have similar addiction mechanisms.

According to three key factors, foreign research suggests a link between adolescent resilience, psychological health, and mobile phone dependence, which has argued that the relationship between college students' resilience and mental health is mediated by Internet addiction, and that increasing resilience helps prevent Internet addiction and reduce the risk of depression [22]. Domestic studies have also shown that mobile phone dependence has a direct effect on college students' physical and mental health, and can also indirectly affect their health through resilience [23]. Internet addiction predicts depression and anxiety in Chinese rural left-behind children, and resilience plays an independent mediating role in the relationship between their Internet addiction and depression and anxiety symptoms [14]. Adverse mental health conditions such as depression, anxiety, stress, and coping styles significantly influence the risk of mobile phone dependence among adolescents and mediate the relationship between resilience and mobile phone dependence among Chinese adolescents [24]. Research has been conducted, albeit sporadically, to support the mechanism by which resilience and mental health work together to influence behavioural problems in mobile phone dependence. Network analyses can further validate this systemic structure.

The family is a direct and dominant subsystem influencing adolescent development, so a harmonious family atmosphere is essential for the healthy physical and mental development of adolescents. During puberty, there is an increase in conflict and a decrease in interaction in parent–child relationships [25]. A Comparative Study of Parent–Child Relationships in the Internet Age in China, the United States, Japan, and Korea showed that 82.1% of Chinese primary and secondary school students had conflicts with their parents, and 25.2% of these conflicts were focused on Internet access. Studies have shown that the parent–child relationship is an important mediating mechanism in the family system that influences individual development and adaptation [26]. Conflict is an important part of the parent–child relationship, and adolescents with higher parent–child conflict are more likely to develop mobile phone dependence [27]. Substantial empirical studies have also demonstrated that parent–child conflict can negatively predict adolescent mental health [28]. In addition, there is an association mechanism between parent–child relationships (including parental support and parent–child conflict) and adolescent resilience [29]. According to the individual-situation interaction theory, the situational factors of parent–child conflict may interact with the resilience of individuals' psychological traits to influence individuals' psychological behavioural states. Therefore, this study will also study the relationship between parent–child conflict and left-behind adolescents' resilience, mental health, and mobile phone dependence.

At present, most domestic and international studies related to resilience, mental health, mobile phone dependence, and parent–child conflict adopt cross-sectional empirical research methods to explore the predictive mechanisms by constructing structural equation modelling [22–24]. While this approach can also deal with relationships between multivariate variables, it is mainly applied to validation factor analyses, focusing on verifying the pre-determined model structure, may have limitations for highly complex and dynamic systems, and may be insensitive to the discovery of new structures and patterns. Resilience [20] and good parent–child relationships [26] are protective factors in children's growth. ‘Protective model’ of adolescent development proposed by Fergus et al. [30], suggests that different protective factors may interact in predicting developmental outcomes, i.e., the predictive effect of one protective factor (e.g., resilience) on outcome variables (e.g., mobile phone dependence, mental health) may be influenced by another protective factor (e.g., parent–child relationship).

In recent years, the network analysis model, as a complement to the latent variable model, has rapidly become a new approach to describing the psychological characteristics of individuals, providing new ideas for understanding human psychological phenomena. In response to the neglect of symptom interactions in the latent variable model from a traditional psychological perspective [31], Borsboom proposed the network theory of psychopathology, which suggests that symptoms are an integral part of mental disorders, and that the onset and persistence of mental disorders are driven by tightly intertwined causality and mutually reinforcing feedback mechanisms between symptoms [32]. Based on this theory, a study by Cramer et al. used a Gaussian graph theoretical model to analyse the relational network of symptoms [33]. Subsequently, the model became the basis of a methodology for processing cross-sectional data using network analysis. The method considers symptoms as nodes of a network graph, links between symptoms as edges connecting the nodes, and the weights of the edges represent the strength of the associations between the nodes, usually visualised as the thickness of the edges in the network graph. Network analysis methods can deal with complex interactions and dynamic relationships between variables and can reveal underlying structures and patterns in a system. Resilience, mental health and mobile phone dependence typically involve multidimensional constructs (e.g., emotional, behavioural, cognitive, etc.), and network analysis can reveal the interactions between these dimensions rather than just aggregating them into a single latent variable. Compared to structural equation modelling, network analysis can not only identify direct interactions between variables, but also reveal the central role of key nodes in the system, providing new ideas for the precise design of interventions.

Therefore, this study takes the group of left-behind adolescents in Sichuan Province, China, as the research object, and explores the multidimensional relationship between resilience, psychological health, and mobile phone dependence through network analysis, and assesses the characteristics of the network structure under different levels of parent–child conflict. This study is geographical and population-specific, combining psychology, sociology, and complex network analysis, which provides a novel theoretical framework and methodological tool for the study of the relationship between resilience, psychological health, and mobile phone dependence among left-behind adolescents, which can help to provide a scientific basis for the development of precise social intervention strategies.

## Methodology

### Participants

This study used an online questionnaire platform to conduct a survey in 28 secondary schools in Sichuan Province for those who met the following criteria: (1) students in their first to third year of high school; (2) fulfilled the condition of being left behind, ‘neither parent can supervise or take care of me’; (3) gave informed consent and voluntarily took part in this research. A total of 2,824 questionnaires were distributed, excluding duplicates, missing questions, and consecutive cases with the same answers, and removing outliers according to the standard deviation of three times, resulting in 2,100 valid questionnaires, with an effective recovery rate of 74.4%.

### Measurement tools

#### Mental health

This study used the Brief Symptom Inventory 18 (BSI-18) prepared by Derogatis [34] to measures mental health. The reliability and validity of the scale have been verified in Chinese adolescents [35]. The scale consists of 18 questions with three dimensions, somatization, depression, and anxiety, and three subscales with six items each. All questions are scored on a 5-point scale (1 = never, 2 = mild, 3 = moderate, 4 = quite severe, 5 = severe), with higher scores indicating higher levels of psychological distress and lower levels of mental health. In this study, Cronbach's alpha coefficient for this scale was 0.926, with 0.845 for the somatization subscale, 0.850 for the depression subscale, and 0.846 for the anxiety subscale.

#### Mobile phone dependence

The Chinese version of the Mobile Phone Addiction Index (MPAI) developed by Leung et al. [6] to measure mobile phone dependence. Previous studies have applied this scale to adolescents in rural China [36]. The scale consists of 17 questions, including four dimensions, namely, the inability to control cravings subscale, the feeling anxious and lost subscale, the withdrawal and escape subscale, and the productivity loss subscale, with the number of questions in each dimension ranging from 3–7. A 5-point scoring system was applied, with higher scores indicating higher levels of individual cell phone addiction. In this study, the Cronbach's alpha coefficient of the scale was 0.892, with 0.849 for the inability to control cravings subscale, 0.786 for the withdrawal and escape subscale, 0.764 for the feeling anxious and lost subscale, and 0.755 for the productivity loss subscale.

#### Resilience

The Resilience Scale for Chinese Adolescents (RSCA) developed by Yue-Qin Hu and Yi-Qun Gan was used in this study [13] to measure resilience. The scale has good reliability and validity and is widely used in Chinese left-behind children [37]. The scale consists of 27 questions, including five dimensions: goal planning, emotional control, positive thinking, family support, and interpersonal assistance, with the number of questions in each dimension ranging from four to six. A 5-point scoring system was used, with higher scores indicating higher levels of resilience. In this study, the Cronbach's alpha coefficient of the scale was 0.874, with 0.763 for the goal-focused subscale, 0. 747 for the emotional control subscale, 0.762 for the positive thinking subscale, 0.793 for the family support subscale, and 0.745 for the interpersonal assistance subscale.

#### Parent–child conflict

Based on Nelissen [38] 's study, the Parent–Child Conflict Scale consists of 6 questions on a 5-point scale, with higher scores indicating higher levels of parent–child conflict. The Cronbach's alpha coefficient for the scale in this study was 0.797.

### Data analysis

In this study, SPSS23.0 was applied for total score calculation, common method bias test, and descriptive statistical analysis, R (4.3.2) was used for network analysis, and R packages qgraph (1.9.8), mgm (1.2–14), networktools (1.5.2), and bootnet (1.5.6) were used for network estimation and visualisation, network centrality estimation and stability tests [39]. And compared using the R package NetworkComparisonTest (2.2.2) [40].

#### Data pre-processing

Invalid questionnaires were filtered according to the following steps: firstly, questionnaires with missing items were deleted to facilitate subsequent data analysis. Then duplicate cases were identified and deleted based on information such as IP, time of submission, age, school, etc. And finally, the Z-scores of each scale and its dimension scores were calculated, and the data of extreme cases with Z-scores exceeding plus or minus 3 were deleted [41] to make the results more stable and reliable.

#### Network estimation and visualisation

For the network with 12 nodes, 66 parameters [12 × (12–1)/2] need to be estimated [39], and according to at least 3–5 individuals per parameter, the sample size is sufficient for network analysis (*N* = 2100). In this study, each dimension of the Brief Symptom Scale 18, the Mobile Phone Dependence Index, and the Resilience Scale for Chinese Adolescents was used as a node, and the correlation between the dimensions were used to generate the edges of the network, and the partial correlation structured network was constructed and visualised using the R package qgraph (1.9.8). Applying the least absolute shrinkage and selection operator (LASSO) and the extended Bayesian information criterion (EBIC). Regularisation was performed with a tuning parameter of 0.5 to prevent overfitting and obtain a concise and interpretable structure. The predictability of each node was calculated using the R package mgm(1.2–14). In addition, using the spinglass algorithm for modular analysis of node clustering to reveal and optimise the structure of associations in the network.

In the network, green edge lines represent positive correlations and red edge lines represent negative phases, and the thickness of the edges indicates the absolute magnitude of the correlation, with thicker edges indicating higher correlations. Using the Fruchterman-Reingold algorithm, which is a visual network layout was performed so that nodes with strong and numerous connections were located in the centre of the network. When performing network comparisons between the high and low level groups of parent–child conflict, the averageLayout function in the R package qgraph (1.9.8) was used to perform network layouts, presenting a consistent visual layout of nodes using the average position in the two networks.

#### Centrality estimates

In network analysis, the centrality metric is an important metric used to describe how central a node is in the network. Using the centrality function in the R package qgraph (1.9.8) to calculate the centrality of the network nodes and use the bridge function in the R package networktools (1.5.2) to calculate bridge centrality for network nodes. As strength centrality has been found to be the most persuasive indicator in psychometrics based on previous research [42], the strength of the nodes and the bridge strength centrality were chosen to be reported in this study and normalised (z-scored) values for each node were plotted.

#### Accuracy and stability test

The accuracy and stability of the constructed network were calculated and verified using the R package bootnet (1.5.6) using the Bootstrapping method. The estimation results were validated and analysed. Firstly, the 95% confidence interval (CI) of each edge weight in the network are calculated based on the non-parametric bootstrapping method. Secondly, based on removing the case-dropping subset bootstrap to assess the stability of the central indicator. And the correlation stability coefficient (CS-coefficient) of the network is calculated using the corStability function for assessment, which higher than 0.5 indicates good centrality stability [39]. Finally, the centrality difference test was conducted to assess the differences in centrality indicators between nodes and edges using the non-parametric bootstrap method. *p* < 0.05 was considered statistically significant.

#### Network comparison

The top 27% of the total parent–child conflict score was taken as the high level of the parent–child conflict group, and the bottom 27% of the total score was taken as the low level of the parent–child conflict group [43], constructed the networks separately. Using the R package NetworkComparisonTest (2.2.2), Network Comparison Test was performed on the high and low-level groups of parent–child conflict. Network invariance test and global strength invariance test were performed in 5000 permutations to assess whether the two networks differed in weight of edges and global strength.

## Results

### Common method bias test

As all data in this study were collected using participant self-report, there may be common methodological bias. The Harman one-way method of testing was used in this study, which showed that there were a total of 12 factors with eigenvalues greater than 1, the first of which had a percentage of the variance of 23.4%, which did not exceed the critical value of 50% [44]. Therefore, common method bias had little effect on the results of this study.

### Descriptive statistics

Of the total of 2,100 adolescent secondary school students included in this study (Table 1), 905 (43.1%) were boys, and 1,195 (56.9%) were girls, with an average age of 15.60 years (age 12 to 20, SD = 1.78); the urban population was 386 (18.4%), the township population was 528 (25.1%), and the rural population was 1,186 (56.5%). In terms of gender, the Cohen's d effect size on the research variables was small for both male and female groups in the sample (Cohen's d: 0.09 ~ 0.16), indicating that gender differences had little effect on the research variables. As far as age is concerned, the correlation between age and the research variables in the sample is low (|r|: 0.08 ~ 0.13), indicating that age has limited effect on the research variables. As for residency, the ANOVA results for the three groups in the sample on the study variables were not significant (*p* > 0.05), indicating that the effect of residence on the study variables was not significant. Specific statistics on participants' left-behind status are detailed in Table 1.

**Table 1:** Basic information of participants

Variable	Number (%) / M (SD)		
	**Synthesis**	**High-level Parent–Child Conflict Group**	**Low-level Parent–Child Conflict Group**
**Gender**			
Boys	905 (43.1%)	219 (38.6%)	290 (51.1%)
Girls	1,195 (56.9%)	348 (61.4%)	277 (48.9%)
**Age**	15.60 (1.78)	15.94 (1.81)	15.30 (1.83)
**Residency**			
Urban	386 (18.4%)	111 (19.6%)	91 (16.0%)
Town	528 (25.1%)	151 (26.6%)	134 (23.6%)
Rural	1,186 (56.5%)	305 (53.8%)	342 (60.3%)
**Living with my father or not**			
Living with my father lack of his custody and care	78 (3.7%)	27 (4.8%)	16 (2.8%)
Within 3 months of my father's absence annually	75 (3.6%)	36 (6.3%)	16 (2.8%)
Within 3–6 months of my father's absence annually	415 (19.8%)	153 (27.0%)	89 (15.7%)
More than 6 months of my father's absence annually	722 (34.4%)	168 (29.6%)	213 (37.6%)
Almost a whole year of my father's absence	754 (35.9%)	172 (30.3%)	217 (38.3%)
My father has passed away	56 (2.7%)	11 (1.9%)	16 (2.8%)
**Living with my mother or not**			
Living with my mother lack of his custody and care	135 (6.4%)	44 (7.8%)	23 (4.1%)
Within 3 months of my mother's absence annually	103 (4.9%)	47 (8.3%)	19 (3.4%)
Within 3–6 months of my mother's absence annually	391 (18.6%)	156 (27.5%)	94 (16.6%)
More than 6 months of my father's absence annually	691 (32.9%)	143 (25.2%)	200 (35.3%)
Almost a whole year of my mother's absence	737 (35.1%)	169 (29.8%)	214 (37.7%)
My mother has passed away	43 (2.0%)	8 (1.4%)	17 (3.0%)

The high level of parent–child conflict group consisted of 567 individuals, 219 males (38.6%) and 348 females (61.4%), with a mean age of 15.94 years (age 12 to 20, SD = 1.81); the low level of parent–child conflict group consisted of 567 individuals, 290 males (51.1%) and 277 females (48.9%), with a mean age of 15.30 years (age 12 to 20, SD = 1.83). Comparing the differences between the high level of parent–child conflict group and the low level of parent–child conflict group on some demographic variables, the descriptive statistics showed that the two groups were essentially similar on the variables of gender, age, residency, and left-behind status (see Table 1). Through t-tests, there were no statistically significant differences (*p* > 0.05) in demographic variables other than age, town and rural residency, suggesting that in most respects the two groups were essentially equivalent.

The overall sample mean total score for the BSI-18 was 33.29 (SD = 10.70, skewness = 0.66, kurtosis = −0.36), the MPAI mean total score was 48.67 (SD = 12.18, skewness = 0.01, kurtosis = −0.08), the RSCA mean total score was 87.43 (SD = 14.61, skewness = 0.22, kurtosis = −0.03), and the Parent–Child Conflict Scale mean total score was 9.66 (SD = 3.42, skewness = 1.08, kurtosis = 0.38). The mean, standard deviation, skewness and kurtosis of each dimension of the scale, i.e., network nodes, are shown in Table 2. The mean of the total score of the BSI-18 for the high level of parent–child conflict group was 41.73 (SD = 10.55, skewness = −0.05, kurtosis = −0.68), the mean of the total score of the MPAI was 52.60 (SD = 11.19, skewness = 0.05, kurtosis = −0.18), the mean of the total score of the RSCA was 77.99 (SD = 12.29, skewness = 0.15, kurtosis = 0.09), and the mean of the total score of the Parent–Child Conflict Scale was 14.38 (SD = 2.54, skewness = 0.40, kurtosis = −1.16). The low level of parent–child conflict group had a total score mean of 26.58 (SD = 7.28, skewness = 1.52, kurtosis = 2.97) for the BSI-18, 42.41 (SD = 11.48, skewness = 0.10, kurtosis = −0.25) for the MPAI, 97.53 (SD = 13.99, skewness = 0.10, kurtosis = −0.55) for the RSCA, and 6.38 (SD = 0.49, skewness = 0.48, kurtosis = −1.77) for the Parent–Child Conflict Scale.

**Table 2:** Mean, standard deviation, skewness, kurtosis, predictability, and centrality indicators for each node

Nodes	Content	Mean (M)	Standard deviation (SD)	Skewness	Kurtosis	Predictability	Strength	Bridge Strength
BSI1	Somatisation	9.96	3.80	0.96	0.08	0.49	0.88	0.37
BSI2	Depression	11.65	4.14	0.58	−0.58	0.64	1.10	0.48
BSI3	Anxiety	11.68	4.16	0.57	−0.58	0.68	1.23	0.45
MPAI1	Inability to control cravings	20.05	6.04	0.01	−0.31	0.51	1.21	0.64
MPAI2	Feeling anxious and lost	10.39	3.67	0.33	−0.20	0.49	1.05	0.34
MPAI3	Withdrawal and escape	8.95	2.95	−0.08	−0.53	0.28	0.68	0.12
MPAI4	Productivity loss	9.28	2.85	−0.21	−0.41	0.39	1.09	0.32
RSCA1	Goal planning	16.26	3.81	−0.06	−0.20	0.35	0.79	0.16
RSCA2	Affect control	18.03	4.27	0.04	−0.09	0.42	0.90	0.49
RSCA3	Positive thinking	14.06	3.17	−0.38	−0.20	0.31	0.88	0.33
RSCA4	Family support	19.95	4.87	−0.22	−0.32	0.45	0.95	0.60
RSCA5	Help-seeking	19.13	4.83	−0.02	−0.22	0.23	0.57	0.24

### Network structure

**Fig. 1:**
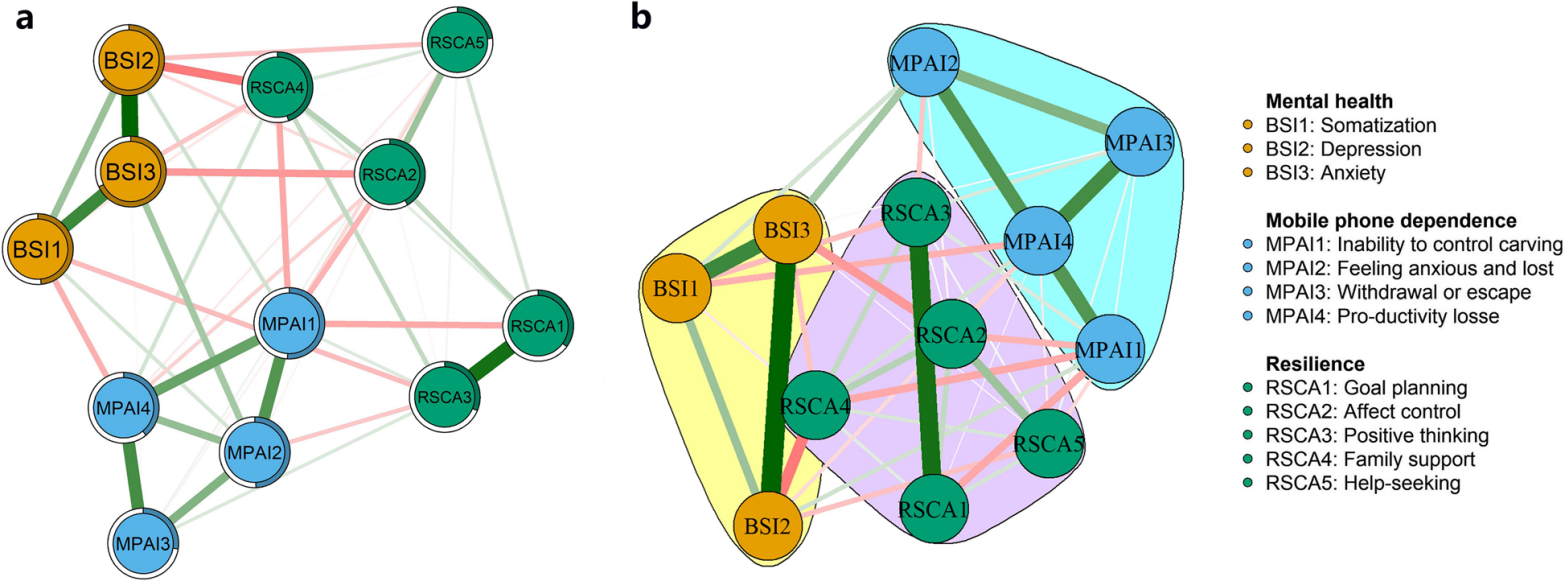
Network structure diagram (**a**) and cluster diagram (**b**) of mobile phone dependence, mental health and resilience among left-behind adolescents. Note: Nodes are psychological variable dimensions, and connecting lines are partial correlations between node dimensions. The thicker the line, the higher the correlation, and the colour of the line indicates the direction of the correlation (green for positive correlation, red for negative)

The network structure of the left-behind adolescents' mental health, mobile phone dependence, and resilience is demonstrated in Fig. 1a. There are 12 nodes in the network, and a total of 45 non-zero edges actually exist, including 20 negative edges and 25 positive edges, accounting for 68.18% of the number of possible connected edges. The proportion of the circle around a node that is filled represents the predictability of that node, with a larger proportion of the filled portion indicating a higher predictability of that node, with an average predictability of 0.44 (range 0.23 to 0.68, Table 2).

The network module analysis displayed that the nodes of mental health, mobile phone dependence, and resilience of the left-behind children clustered with each other to form three node communities (Fig. 1b), which was consistent with the three research variables and their dimensions. The communities for mental health and mobile phone dependence were more strongly connected internally; whereas the communities for resilience were weaker except for RSCA1 (goal planning) and RSCA3 (positive thinking) which were deeply associated. The links between the three communities were also stronger, with the dimensional nodes interacting with each other. The strongest connections were between BSI2 (depression) and RSCA4 (family support), BSI3 (anxiety) with RSCA2 (affect control) and MPAI2 (the feeling anxious and lost), and MPAI1 (inability to control cravings) with RSCA4 (family support) and RSCA1 (goal planning) directly.

### Indicators of centrality

**Fig. 2:**
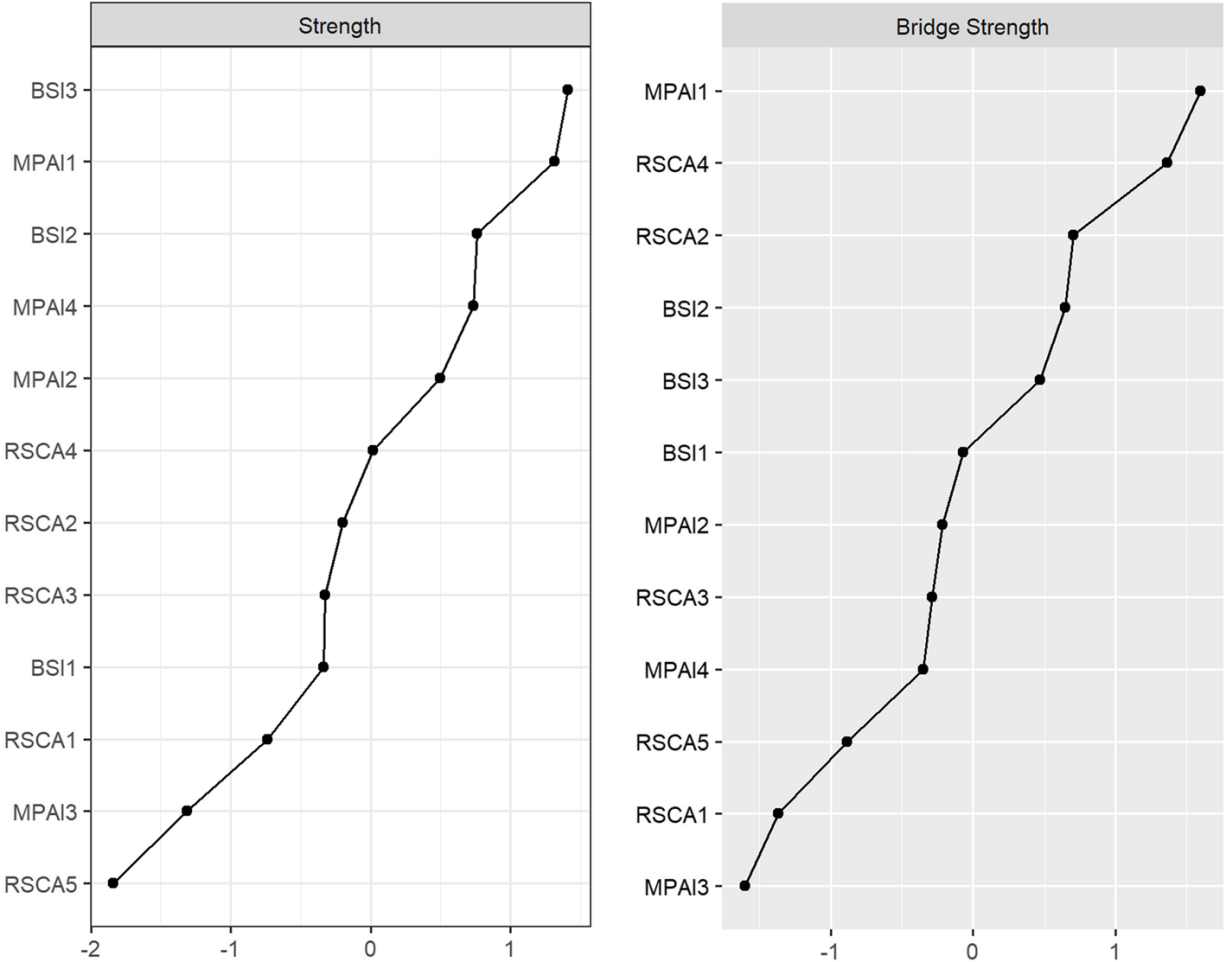
Centrality index of each node of the network. Note: BSI1, somatization; BSI2, depression; BSI3, anxiety; MPAI1, inability to control carving; MPAI2, feeling anxious and lost; MPAI3, withdrawal or escape; MPAI4, productivity losse; RSCA1, goal planning; RSCA2, affect control; RSCA3, positive thinking; RSCA4, family support; RSCA5, help-seeking

The results of the centrality index of psychological status, mobile phone dependence and resilience network of left-behind adolescents are shown in Fig. 2, and the specific values are shown in Table 2. Node BSI3 (anxiety, Strength = 1.23) has the highest strength centrality, and node MPAI1 (inability to control cravings, Strength = 1.21) is the second highest. In terms of bridge strength centrality, nodes MPAI1 (inability to control carving, Bridge Strength = 0.64) and RSCA4 (family support, Bridge Strength = 0.60) were significantly stronger than the other nodes. The results of the variability test for the centrality index also indicate that the high centrality nodes are stable and reliable.

### Accuracy and stability of the network

**Fig. 3:**
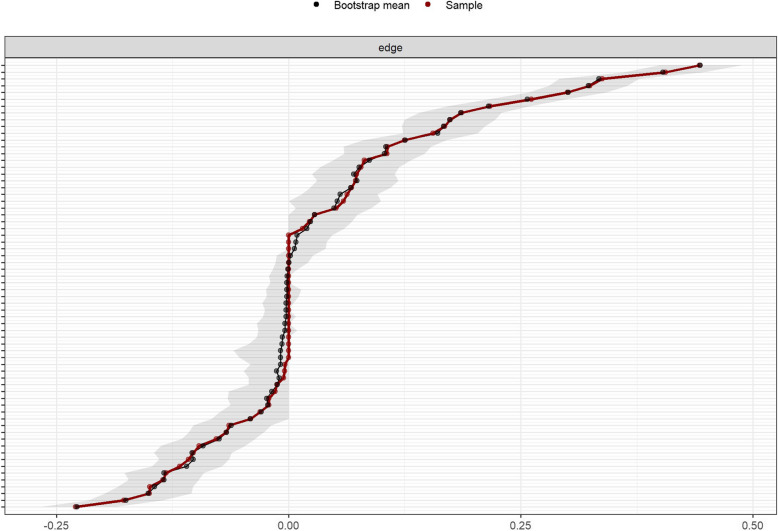
Bootstrap confidence intervals for edge weights in network. Note: Red dots indicate sample values, black dots indicate values for each edge weight, and grey areas indicate 95% confidence intervals

**Fig. 4:**
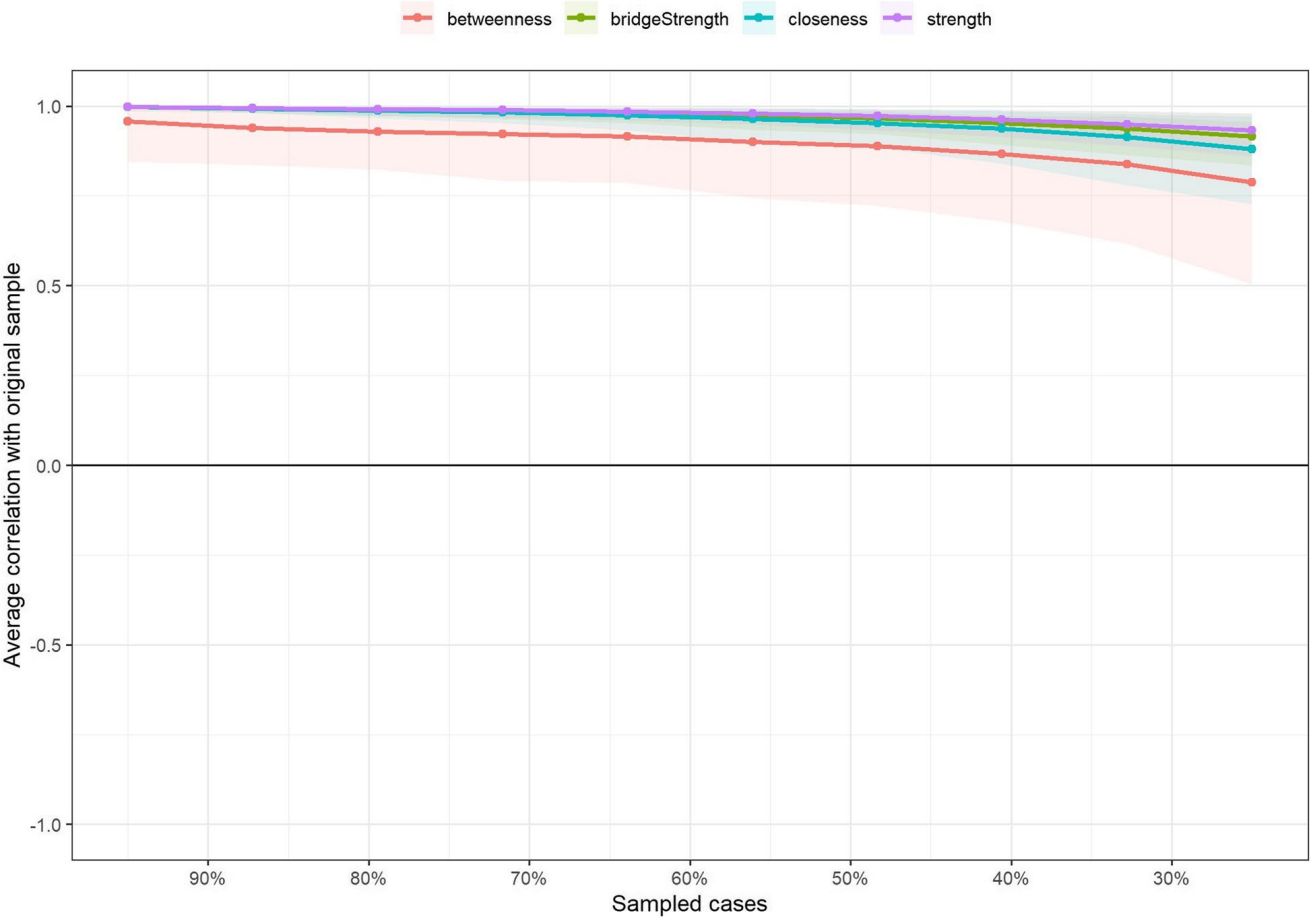
Stability test diagram for the case-dropping subset. Note: Lines represent the average relationship between the original sample centrality and the subsamples. Regional color blocks indicate the range between the first quartile and the third quartile

**Fig. 5:**
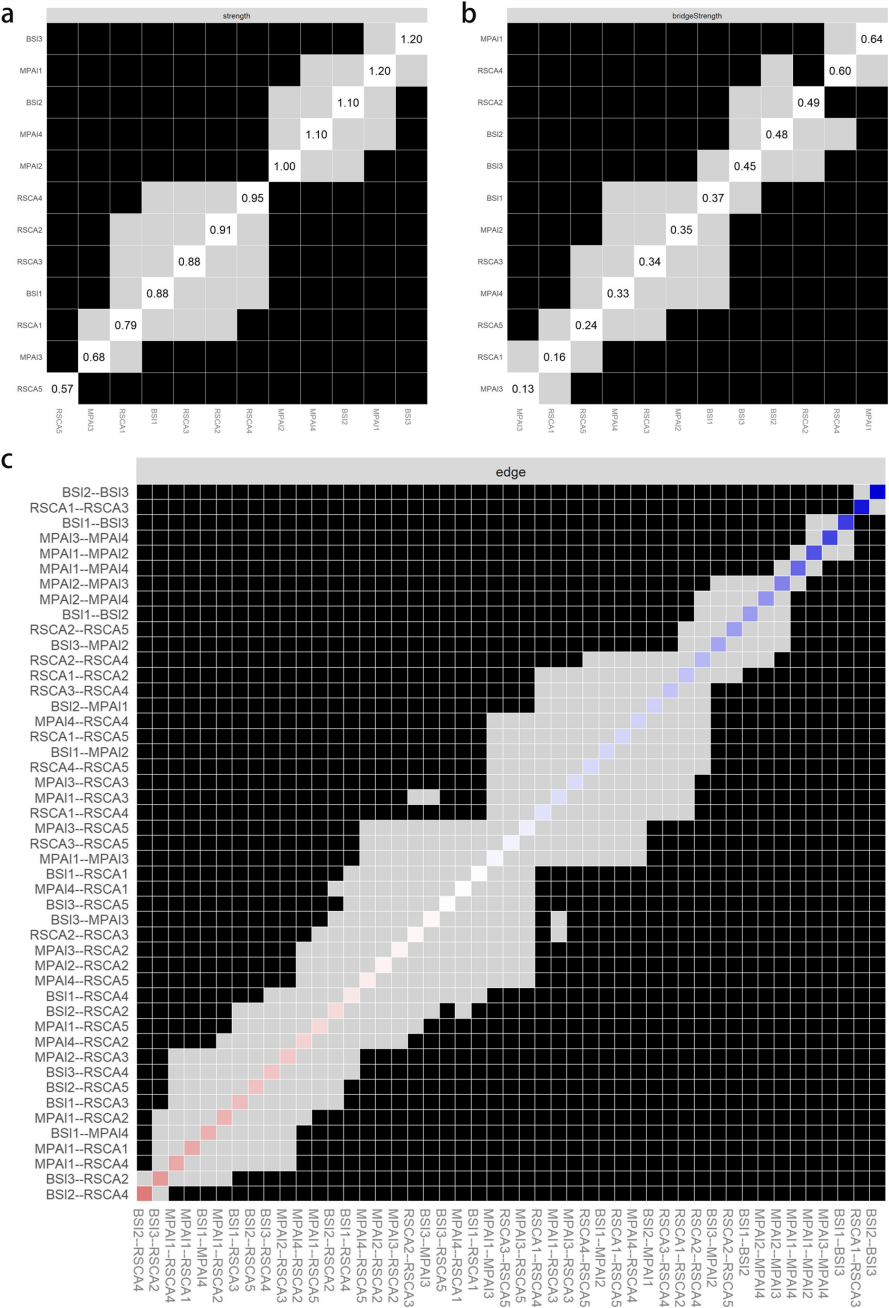
Bootstrapped difference tests result of the network strength (**a**), bridge strength (**b**) and edges (**c**). Note: Black boxes indicate significant differences between two nodes (α = 0.05). BSI1, somatization; BSI2, depression; BSI3, anxiety; MPAI1, inability to control carving; MPAI2, feeling anxious and lost; MPAI3, withdrawal or escape; MPAI4, productivity losse; RSCA1, goal planning; RSCA2, affect control; RSCA3, positive thinking; RSCA4, family support; RSCA5, help-seeking

The results of the edge weight bootstrap procedure are shown in Fig. 3, where the network estimation is moderately accurate and there is a partial overlap between the 95% CI of the edge weights. The results of the excluded cases bootstrap method are shown in Fig. 4, with CS coefficients of 0.75 for strength, bridge strength, closeness, and edges, and 0.594 for betweenness, which are greater than 0.5, saying that the network estimation has good stability. The result of bootstrapped difference tests is shown in Fig. 5, nodes and edges with strong centrality in the network are statistically more strongly different than other nodes in the network, further indicating that the results of centrality analysis are stable and generalisable.

### Comparison of networks

**Fig. 6:**
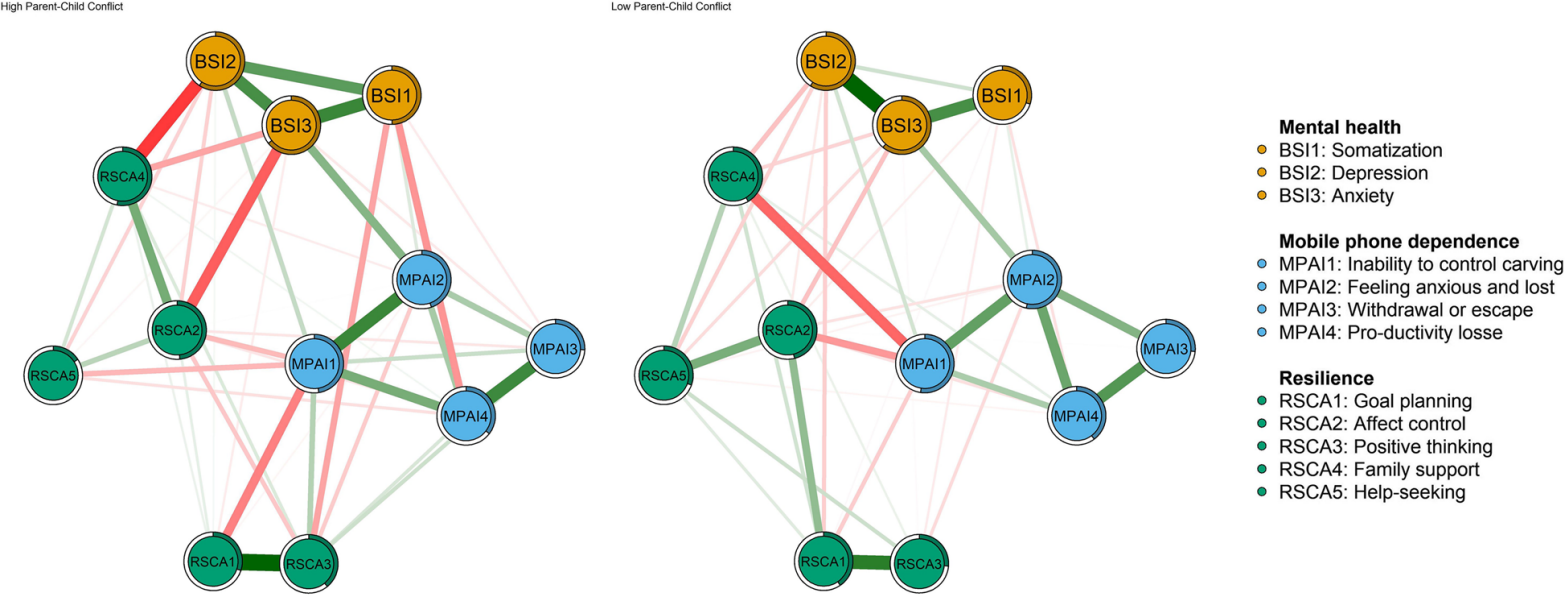
Comparison of network structure between high- and low-level groups of parent–child conflict. Note: Nodes are dimensions of psychological variables, and lines are partial correlations between the dimensions of nodes. Thicker lines indicate higher correlation, and line colors indicate the direction of correlation (green for positive correlation, red for negative correlation)

**Fig. 7:**
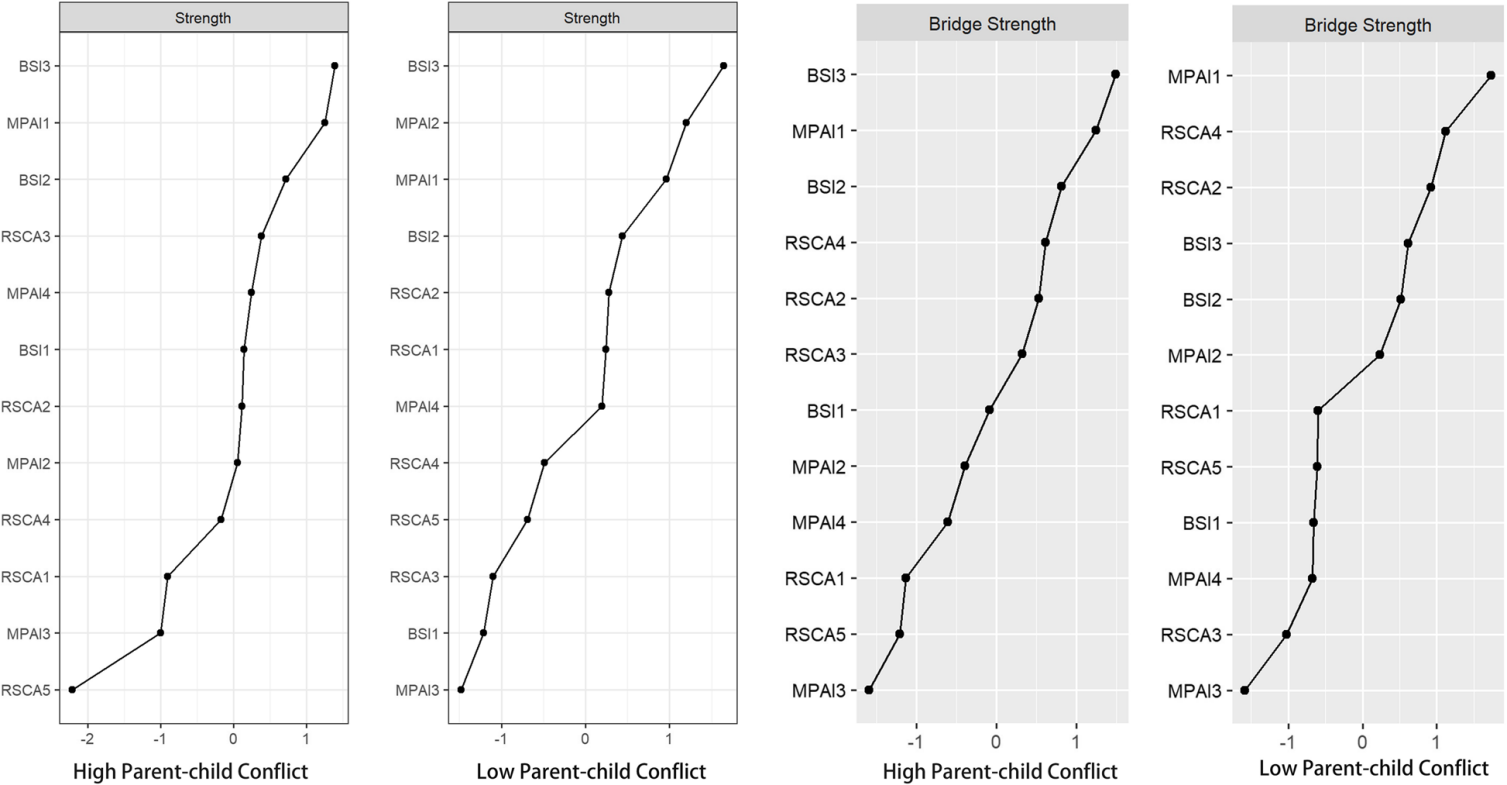
Comparison of network centrality indicators between high- and low-level groups of parent–child conflict. Note: The centrality graph depicts the strength centrality (z-score) and the bridge strength centrality (z-score) of each node in the network, with higher scores representing greater influence of nodes in the network. BSI1, somatization; BSI2, depression; BSI3, anxiety; MPAI1, inability to control carving; MPAI2, feeling anxious and lost; MPAI3, withdrawal or escape; MPAI4, productivity losse; RSCA1, goal planning; RSCA2, affect control; RSCA3, positive thinking; RSCA4, family support; RSCA5, help-seeking

Network Comparison Test (NCT) was performed on the Parent–Child Conflict High-Level Group and Low-Level Group. The results show that both networks contain 12 nodes, and the result of the parent–child conflict high-level group contains 47 edges, while the parent–child conflict low-level group contains 42 edges, and the visualisation of the network is shown in Fig. 6. Families with high levels of parent–child conflict were more connected within the mental health and mobile phone dependence communities, and distant within the resilience community, and even showed a significant negative internal correlation. In addition, the network structure of the high-level parent–child conflict group had stronger direct associations among the three communities. In contrast, in the group with low levels of parent–child conflict, inter-community connections were relatively looser, intro-community associations within resilience were stronger, and the direct link between mental health and mobile phone dependence communities was significantly reduced. By centrality analysis, in the parent–child conflict high-level group, the core nodes and core bridge nodes are BSI3 (anxiety) and MPAI1 (inability to control cravings). In the low-level group of parent–child conflict, the core nodes were BSI3 (anxiety), MPAI2 (the feeling anxious and lost), and the core bridge nodes were MPAI1 (inability to control cravings), and RSCA4 (family support). The detailed network centrality is shown in Fig. 7.

The results of the network invariance test showed a significant difference in structure between the high and low-level groups of parent–child conflict (M = 0.265, *p* < 0.001), and the results of the global strength invariance test did not find a significant difference in the global strength of the network (high-level group: 5.526 vs. low-level group: 4.952; S = 0.566, *p* = 0.162). Tests of centrality invariance revealed significant differences in both intensity centrality (*p* < 0.001, cohen's d = 0.456) and bridge intensity centrality (*p* < 0.001, cohen's d = 0.828). A total of 6 node centrality of BSI1 (somatization), RSCA5 (interpersonal assistance), and BSI2 (depression) were significantly different (*p* < 0.05), accounting for 50% of the total. The results of the borderline weight invariance test showed that a total of 15 borderlines such as RSCA4 (family support) differed significantly (*p* < 0.05) from BSI2 (depression), MPAI1 (inability to control cravings), and RSCA2 (affect control), and BSI1 (somatization) differed significantly (*p* < 0.05) from BSI2 (depression), and MPAI4 (productivity loss), which accounted for about 28% of the total number of borderlines.

## Discussion

In this study, we used network analysis to explore in depth the associations between the dimensions of resilience and mental health and mobile phone dependence among Chinese left-behind adolescents, and to further compare the core dimensions and network structure differences in the networks of resilience and mental health and mobile phone dependence among left-behind adolescents with different levels of parent–child conflict. The results of the study found that: (1) there exists a structurally stable network relationship between resilience, psychological health, and mobile phone dependence among left-behind adolescents; (2) BSI3 (anxiety) is the most central node in the network model, followed by MPAI1 (inability to control cravings); (3) MPAI1 (inability to control cravings) and RSCA4 (family support) are the most central bridge nodes connecting resilience, psychological health and mobile phone dependence in the study sample; (4) there were significant differences in the network structure between the high and low-level groups of parent–child conflict, no significant differences in the global strength of the network, and significant differences in both strength centrality and bridge strength centrality.

### Network structure and its core dimensions of resilience, mental health, and mobile phone dependence of left-behind adolescents

#### Network structure

The study showed that there were three relatively independent clusters in the networks of resilience and mental health and mobile phone dependence among left-behind adolescents. The mental health and mobile phone dependence communities are more closely connected internally, in line with psychopathology network theory [32], i.e. some symptoms are more closely connected to each other than others, and the clusters form manifestations of mental disorders. Connections within the resilience community are generally weaker, with a notable strong positive correlation observed between goal planning and positive thinking. The five dimensions represent various aspects of the individual and their environment, collectively contributing to the overall resilience of the person. When examining the network as a whole, mental health and mobile phone dependence display strong negative associations with resilience, while mental health and mobile phone dependence are positively correlated with each other. Strengthening resilience may help reduce the risk of mobile phone dependence and improve mental health among left-behind adolescents.

#### Network core dimensions

In this study, inability to control cravings was found to be both a core node and a core bridge node in the overall network, manifesting itself in individuals' difficulties in self-regulation and investing too much time in mobile phone use to manage it effectively [6]. The results of this study are similar to those of existing network analysis studies [45]. Inability to control cravings creates an attentional bias towards the automatisation of the addictive substance [46], which affects goal planning such as the allocation of attention and the use of cognitive resources by the dependent person. According to the social surrogacy hypothesis, addiction to smartphones can neglect face-to-face interactions with friends and family and a lack of real-world support. And left-behind adolescents are inherently more deprived of parental companionship, leading to lower levels of family support and interpersonal assistance. For mental health, uncontrollability was only directly related to depression. This may be due to the fact that both mobile phone dependence and depression are related to the dopamine system in the brain [47, 48], and both show similar symptoms such as loss of interest, social withdrawal, and mood swings [49]. Notably, the results of the present study showed that inability to control carving in mobile phone dependence was positively associated with positive thinking of resilience, in contrast to existing research where hopeful attitudes may reduce adolescents' dependence on smartphones [50]. Left-behind adolescents may need to take on family responsibilities earlier because of the unique nature of their home environment, an experience that may allow them to hone their independence and coping strategies [51]. And thus be able to adapt to adversity more quickly in certain situations, and to make self-determination, self-planning and problem-solving with a more optimistic and positive attitude. This also suggests that left-behind adolescents may realise the seriousness of the problem after experiencing uncontrolled mobile phone dependence, and may engage in self-reflection and seek adjustment, in which their positive thinking may be enhanced.

Furthermore, consistent with previous network analysis studies [52], anxiety was likewise a central node in the network model of this study. Anxiety is the brain's response to danger, stimuli, and is a state that an organism will actively try to avoid [34]. The excessive reassurance pathway in the pathway model of problematic mobile phone use proposed by Billieux [53] states that individuals with increased anxiety contribute to their mobile phone dependence addiction out of needs such as comforting reassurance. Meanwhile, according to the Attention-Gate Model (AGM), anxiety reinforces attention to negative stimuli and may lead individuals to overestimate the time interval between negative events. When mobile phone dependent individuals are anxious, their attention may be focused on the time they are waiting to use their mobile phones, which in turn creates distortions in time perception and exacerbates the difficulty of withdrawal. Research has also shown that individuals in a chronic state of anxiety are significantly more sensitive to external stimuli [54], which may further hamper the ability to regulate negative emotions. Moreover, in traditional Chinese culture, children are often expected to restrain their emotions and respect their elders, which may lead left-behind adolescents to be more inclined to repress negative emotions than to effectively resolve them. Anxiety may also weaken the individual's perception and utilisation of family support [55], or prevent left-behind adolescents from obtaining necessary support from family members by triggering communication barriers.

Family support is another one of the core bridge nodes in the network structure of this study, refers to the tolerant, respectful and supportive attitudes of family members [13] and is an important external factor in resilience. Within the context of China's collectivist culture, the central values of family responsibility and intergenerational support more profoundly influence the role of family support in resilience. Family support is closely related to the mental health of family members, with the most significant effect on depression in this study. Lack of parental care leading to depression is one of the most prominent problems among left-behind children [56]. The lack of direct parent–child interaction and emotional communication among left-behind adolescents, and the sense of stress resulting from diminished parental care resources or unmet needs have both immediate and delayed negative predictive effects on their depression [57]. In contrast to the findings of previous studies in which family support significantly negatively predicted adolescent mobile phone dependence [27], family support and productivity loss yet showed positively correlated results in the network structure of this study. Productivity loss refers to excessive mobile phone use resulting in lower academic or work productivity [6]. Left-behind adolescents usually grow up with their grandparents, who are more lenient than their parents based on their love for their grandchildren and who do not know how to deal with adolescents' problematic behaviours due to their lack of knowledge and backwardness [58]. Parents who work outside the home tend to feel indebted to their children and indulge them completely, and lack proper guidance and supervision of their children's learning in education management. Such intergenerational education is often prone to spoiling, and although a certain degree of family support is provided for children, improper discipline in life also leads to problematic behaviours of excessive mobile phone use and affects learning efficiency.

The results of this study, especially the findings of anxiety, inability to control cravings and family support as core nodes, can be closely aligned with Richardson's resilience model [20] to provide further theoretical support for understanding the mechanisms of psychological resilience in left-behind adolescents. In Chinese culture, high expectations for children are often regarded as core elements of family education. However, in the context of left-behind adolescents, such expectations may bring about great psychological stress due to over-control, insufficient communication and lack of emotion [59], which in turn become risk factors in the model. In Richardson's model, anxiety corresponds to the ‘Reintegration with Loss’ of individual balance disintegration produced by insufficient protective factors, i.e., when an individual is faced with excessive pressure or stimulation, the original psychological balance is disrupted [20], and anxiety becomes the driving force that prompts the individual to make adjustments. In the further absence of effective protective factors, anxiety may lead to dysfunction, turning to unhealthy ways of temporarily escaping from stress through mobile phone dependence and other unhealthy ways, and entering a lower level of the ‘Dysfunctional Reorganisation’ or even imbalance. In Chinese collectivist culture, family relationships are central to an individual's emotional support, making family support an important protective factor that can provide psychological and behavioural support for individual reintegration.

### Differences in parent–child conflict levels between resilience, mental health, and mobile phone dependence networks in adolescents who are left behind

The results of the network comparison test in this study showed similar levels of overall connectivity in both networks, suggesting a degree of stability across different levels of parent–child conflict. In the high-conflict group, the network displayed more correlated edges, suggesting more complex interactions between psychological problems, resilience, and mobile phone dependence. This finding reflects the idea that intense parent–child conflict can trigger the rapid spread of negative factors while allowing individuals to demonstrate considerable flexibility and diversity in psychological adaptation [60]. Such adaptability enables them to cope with different situations using multiple psychological mechanisms. However, this adaptation can undermine the synergies within psychological resilience. For instance, positive cognitive strategies may be employed more to rationalize the negative family environment than to foster emotional regulation or enhance family support, resulting in weaker or even antagonistic connections between resilience components. Furthermore, traditional Chinese cultural values that emphasize parental authority and harsh parenting styles exacerbate this issue. Parents may suppress adolescents' autonomy and emotional expression during conflicts, making it difficult for them to receive effective support or regulatory resources from their families. This family environment not only weakens the synergy among elements of adolescent resilience but also may intensify the disorder within resilience by disrupting the balance between affect control and positive thinking. These combined stresses make it more difficult for adolescents in conflict-ridden families to develop a stable and strong resilience to cope with crises. In contrast, the networks in low-conflict groups exhibited weaker inter-community associations, reflecting the relative stability of adolescents' psychological states in such environments. Harmonious parent–child relationships enable adolescents to effectively utilize family support as a core resource of psychological resilience, facilitating the integration of various resilience components. In this context, psychological resilience—especially family support—functions as a bridging node that connects mental health and behavioral problems, helping to alleviate negative emotions. This suggests that in low-conflict environments, psychological resilience plays a buffering role in the interaction between psychological problems and mobile phone dependence [14, 23], thereby offering a stronger protective effect.

Somatization was the node with the most significant central difference in intensity, it has been demonstrated that parent-adolescent conflict can exacerbate adolescent somatization symptoms [61]. Parent–child conflict is one of the stressors for adolescents in puberty [62], prolonged exposure to high-pressure and stressful environments increases the risk of mental health problems in adolescents and is more likely to lead to multiple co-morbidities of psychological problems [63]. In the present study, the more severe the somatization, the less inefficiency caused by mobile phone dependence, which may be due to the physical discomfort of somatization and intense parent–child conflict are the more dominant causes of academic inefficiency among left-behind adolescents. It is also possible that the separation anxiety of parents of left-behind adolescents and severe family conflict lead to stricter parental psychological and behavioural control, which prevents excessive mobile phone use from affecting the adolescents' efficiency. Positive thinking was the node with the most significant difference in bridge strength centrality. Networks of high parent–child conflict suggest that more severe smartphone addiction problems such as lack of self-control, escapism, and inefficiency are intertwined with more optimistic attitudes, which is inconsistent with existing research [64]. In addition to the previously mentioned ability of left-behind adolescents to adapt more quickly to adversity, make positive self-decisions and plans, and potentially reflect deeply and seek change, it is also possible that negative cognition allows them to feel the realities of the situation such as family conflict more acutely, rather than being fully immersed in the world of mobile phones, which reduces performance in areas such as loss of control [51]. However, at the same time, according to the ‘loss of compensation’ hypothesis, their addiction to mobile phones may be more of an emotional attachment, and their inner dependence on mobile phones will be strongly manifested during withdrawal. In addition, The connecting line between inability to control carving and family support is borderline with the most significant difference in weights and is strongly negatively correlated in the low-level group. Family members in families with low parent–child conflict tend to use positive coping strategies [65], and family support may be more accessible and effective. In contrast, if the parent–child conflict is high, although family support may be present, its effectiveness may be diminished by the conflict, and adolescents may seek other forms of social support to cope with the conflict, thus attenuating the direct effect of family support on mobile phone dependence loss of control, and instead having interpersonal assistance directly associated with loss of control.

### Research limitations and future research perspectives

This study used a network analysis model to examine the relationship between psychological variables, providing a multidimensional understanding of left-behind adolescents' resilience in relation to mental health, mobile phone dependence, and parent–child conflict from a cross-sectional perspective for psychological research, as well as expanding and deepening the theory of resilience. This study reveals that teachers and clinical interveners can maximise psychosocial interventions by focusing on the high centrality dimensions such as anxiety, inability to control carving, and family support when confronting left-behind adolescents' mental health and mobile phone dependence issues [66]. For the special case of left-behind adolescents, firstly, family support systems can be upgraded through psychosocial interventions or teacher interventions to reduce conflicts. Secondly, schools organise sports [67] and promote peer contact to reduce the frequency of mobile phone use. Further, psychological counselling and relaxation training can be used to help adolescents establish emotional regulation and self-control. Emotional relief, such as mindfulness-based interventions [68] and cognitive reappraisal [69], is provided for high anxiety adolescents. In addition, the potential ability of left-behind adolescents should be paid attention to and stimulated from the perspective of strengths [51], so that life challenges can be transformed into growth opportunities, and the ability to resist setbacks can be comprehensively improved.

However, this study still has some limitations. First, this study only used cross-sectional data to construct a partial correlation network, and was unable to infer causal relationships. Although the important role of core nodes can be affirmed based on the network model centrality feature [66], it should be verified by longitudinal or experimental design in the future. Second, although this study focused on the interaction between resilience, mental health and mobile phone dependence, other possible key variables were not included, which may impose some limitations on the interpretation of the results. Meanwhile, the average predictability of the nodes of the network analysis model in this study was not high, indicating that the networks could not predict each other well internally and were influenced by factors outside the network (e.g., environmental, biological factors, other psychological variables) [70]. Future research can collect more data with more representativeness and accuracy, identify and control for external variables that may affect the predictability of the model, and also incorporate multimodal indicators to construct the network based on relevant theories. Third, mobile phone dependence measured through self-report may be affected by social expectation bias. Some experiments have found that self-reported mobile phone use does not match the actual situation [71], future research should be cautious in interpreting estimated smartphone use with more objective metrics or a combination of personal interviews and guardian observations for evaluation.

## Conclusion

(1) The network structure of left-behind adolescents' resilience, mental health, and mobile phone dependence is stable, in which anxiety and inability to control cravings are core nodes, and controlling inability and family support are core bridge nodes. Practitioners should focus on the high centrality dimension for effective intervention for left-behind adolescents.

(2) There are significant differences in the network structure of resilience, psychological health, and mobile phone dependence among left-behind adolescents at different levels of parent–child conflict. In families with higher levels of parent–child conflict, the network structure is more complex, and the resilience of left-behind adolescents is undermined, with risks and negative effects spreading faster; while in the lower counterpart, resilience has a protective effect.

## Supplementary Information

Supplementary Material 1.

## Data Availability

The data supporting the findings of this study are available from the corresponding author, upon reasonable request, immediately following publication and no end date. We can share individual participant data that underlie the results reported in this article, after deidentification (text, tables, figures and appendices).

## Abbreviations

BSI1: Somatisation
BSI2: Depression
BSI3: Anxiety
MPAI1: Inability to control cravings
MPAI2: Feeling anxious and lost
MPAI3: Withdrawal and escape
MPAI4: Productivity loss
RSCA1: Goal planning
RSCA2: Affect control
RSCA3: Positive thinking
RSCA4: Family support
RSCA5: Help-seeking
BSI-18: Brief Symptom Inventory 18
MPAI: Mobile Phone Addiction Index
RSCA: Resilience Scale for Chinese Adolescents
