# Supplementary material for: The relationship between resilience and mental health: mobile phone dependence and its differences across levels of parent-child conflict among left-behind adolescents: a cross-sectional network analysis
Source: BMC Public Health. 2025 Mar 10;25:940. doi: 10.1186/s12889-025-22105-8 (PMC11892170; doi:10.1186/s12889-025-22105-8)
Supplement: Supplementary file 1 — Supplementary Material 1. [file 12889_2025_22105_MOESM1_ESM.docx]

**Basic Information** **Questionnaire**

Dear students,

Hello! This project is a survey project for adolescents. Your participation and cooperation are very important for us to understand the mental health status of adolescents. Thank you for filling in the following questionnaire. We will keep the information you fill in completely confidential. This questionnaire is for research use only. Thank you very much for your cooperation!

1. Your age is ( ) years old.

2. Your grade is :

□Grade 1 in junior high school

□Grade 2 in junior high school

□Grade 3 in junior high school

□Grade 1 in senior high school

□Grade 2 in senior high school

□Grade 3 in senior high school

3. Your place of residence is :

□In the city

□In the town

□In the countryside

4. Does your father live with you?

□Yes, and can monitor and take care of me

□Yes, but can't monitor and take care of me

□No, generally will be away within 3 months a year

□No, generally will be away for 3-6 months a year

□No, generally will be away for more than half a year

□No, almost all of the year away

□Father died

5. Does your mother live with you?

□Yes, and can monitor and take care of me

□Yes, but can't monitor and take care of me

□No, generally will be away within 3 months a year

□No, generally will be away for 3-6 months a year

□No, generally will be away for more than half a year

□No, almost all of the year away

□Mother died

The above is the self-compiled questionnaire used in this study to collect the demographic information of the participants, and the statistical results are shown in Table 1.

**Questionnaire 1 (BSI-18)**

Mental health was measured using the Brief Symptom Inventory 18 (BSI-18) compiled by Derogati in 2001. Reference have been noted in the manuscript “66. Derogatis LR. BSI 18, Brief Symptom Inventory 18: Administration, scoring and procedures manual. NCS Pearson, Incorporated; 2001. ”. In this study, Cronbach's alpha coefficient for this scale was 0.926. The results are presented in Section 3.2 Descriptive statistics and Table 2 in the manuscript.

**Questionnaire 2 (MPAI)**

The Chinese version of the Mobile Phone Dependence Index (MPAI) developed by Leung et al. in 2008 to measure mobile phone addiction. Reference have been noted in the manuscript “67. Leung L. Leisure boredom, sensation seeking, self-esteem, and addiction. Mediated Interpersonal Communication. 2008;359.” . In this study, Cronbach's alpha coefficient for this scale was 0.892. The results are presented in Section 3.2 Descriptive statistics and Table 2 in the manuscript.

**Questionnaire 3 (RSCA)**

The Resilience Scale for Chinese Adolescents (RSCA) developed by Yue-Qin Hu and Yi-Qun Gan in 2008 was used in this study to measure resilience. Reference have been noted in the manuscript “68. Yue-Qin H, Yi-Qun G. Development and Psychometric Validity of the Resilience Scale for Chinese Adolescents. Acta Psychologica Sinica. 2008 Aug 30;40(08):902. ”. In this study, Cronbach's alpha coefficient for this scale was 0.874. The results are presented in Section 3.2 Descriptive statistics and Table 2 in the manuscript.

**Questionnaire 4**

The Parent-Child Conflict Scale comes from Nelissen's research in 2018 and is used to measure parent-child conflict in this study. References have been noted in the manuscript “69. Nelissen S. The Child Effect in Media Use: Investigating Family Dynamics Concerning Media Behavior in Parent-Child Dyads. 2018; ”. In this study, Cronbach's alpha coefficient for this scale was 0.797. The results are presented in Section 3.2 Descriptive statistics in the manuscript.
